# miR-200a Attenuated Doxorubicin-Induced Cardiotoxicity through Upregulation of Nrf2 in Mice

**DOI:** 10.1155/2019/1512326

**Published:** 2019-11-03

**Authors:** Xiaoping Hu, Huagang Liu, Zhiwei Wang, Zhipeng Hu, Luocheng Li

**Affiliations:** ^1^Department of Cardiovascular Surgery, Renmin Hospital of Wuhan University, Wuhan, Hubei 430060, China; ^2^Department of Vascular Surgery, Renmin Hospital of Wuhan University, Wuhan, Hubei 430060, China

## Abstract

Nuclear factor (erythroid-derived 2)-like 2 (Nrf2) was closely involved in doxorubicin- (DOX-) induced cardiotoxicity. MicroRNA-200a (miR-200a) could target Keap1 mRNA and promote degradation of Keap1 mRNA, resulting in Nrf2 activation. However, the role of miR-200a in DOX-related cardiotoxicity remained unclear. Our study is aimed at investigating the effect of miR-200a on DOX-induced cardiotoxicity in mice. For cardiotropic expression, male mice received an injection of an adeno-associated virus 9 (AAV9) system carrying miR-200a or miR-scramble. Four weeks later, mice received a single intraperitoneal injection of DOX at 15 mg/kg. In our study, we found that miR-200a mRNA was the only microRNA that was significantly decreased in DOX-treated mice and H9c2 cells. miR-200a supplementation blocked whole-body wasting and heart atrophy caused by acute DOX injection, decreased the levels of cardiac troponin I and the N-terminal probrain natriuretic peptide, and improved cardiac and adult cardiomyocyte contractile function. Moreover, miR-200a reduced oxidative stress and cardiac apoptosis without affecting matrix metalloproteinase and inflammatory factors in mice with acute DOX injection. miR-200a also attenuated DOX-induced oxidative injury and cell loss in vitro. As expected, we found that miR-200a activated Nrf2 and Nrf2 deficiency abolished the protection provided by miR-200a supplementation in mice. miR-200a also provided cardiac benefits in a chronic model of DOX-induced cardiotoxicity. In conclusion, miR-200a protected against DOX-induced cardiotoxicity via activation of the Nrf2 signaling pathway. Our data suggest that miR-200a may represent a new cardioprotective strategy against DOX-induced cardiotoxicity.

## 1. Introduction

Doxorubicin (DOX), a quinone-containing anthracycline, has been widely used for the treatment of both solid and hematologic malignancies [1]. Its therapeutic use is limited by its dose-dependent cardiotoxicity, resulting in cardiomyocyte loss, mitochondrial dysfunction, myofibrillar degeneration, and congestive heart failure with poor prognosis [2]. The pathogenesis of DOX-induced cardiotoxicity is complex, but a solid body of evidence indicates that oxidative stress is closely involved [3].

There were a higher level of mitochondria and relatively lower levels of antioxidant enzymes in the heart samples, making the heart more sensitive to DOX-related injury [4]. It has been reported that oxidative stress and subsequent lipid peroxidation could be detected in the hearts within three hours after DOX treatment [5]. Accumulation of reactive oxygen species (ROS) resulted in structural changes of biological macromolecules and death of cardiomyocytes [6]. Therefore, the search for an effective and safe antagonist of oxidative stress would be of great significance for the treatment of DOX-related cardiac toxicity.

Nuclear factor (erythroid-derived 2)-like 2 (Nrf2), a basic leucine zipper transcription factor, is essential for detoxification gene regulation in mammals [7]. Under physiological conditions, Nrf2 is bound in the cytosol by Kelch-like ECH associating protein 1 (Keap1), which is a scaffold protein for the ubiquitination and degradation of Nrf2 [8]. In response to oxidative stress, Nrf2 is released from Keap1 and translocates to the nucleus to regulate the expression of antioxidant and detoxification gene [9]. Nrf2 was decreased in DOX-treated hearts, and restoration of Nrf2 could mitigate DOX-induced cardiotoxicity [10], suggesting targeting Nrf2 as a therapeutic strategy for the treatment of DOX-induced cardiotoxicity.

MicroRNAs (miRNAs or miRs) are highly conserved single-stranded noncoding RNAs that can posttranslationally modify mRNA [11]. Dysregulation of miRNAs has been implicated in many diseases including doxorubicin-induced cardiotoxicity [12]. The miR-200 family has been highlighted for its action in the maintenance of the epithelial phenotype [13]. A recent study indicated that miR-200a acted as a tumor suppressor by targeting FOXA1 in glioma [14]. miR-200a attenuated myocardial necroptosis via targeting RING finger protein 11 [15]. Moreover, miR-200a resulted in Nrf2 activation by targeting Keap1 mRNA and promoting degradation of Keap1 mRNA in diabetic nephropathy [16]. We hypothesized that overexpression of miR-200a in mice could attenuate DOX-induced cardiotoxicity. To investigate this possibility, we overexpressed miR-200a using an adeno-associated virus 9 (AAV9) system in DOX-treated mice. We examined the effect of miR-200a overexpression on DOX-induced oxidative injury and cell apoptosis in mice.

## 2. Methods

### 2.1. Animals

Protocols involving the use of animals were approved by the Institutional Animal Care and Use Committees in Renmin Hospital of Wuhan University. C57BL/6J male mice were also purchased from the Jackson Laboratory and housed in Renmin Hospital of Wuhan University with free access to food and water. An AAV9 system carrying miR-200a or miR-scramble was generated by Hanbio Technology (Shanghai, China). For cardiotropic expression, male mice (age: 8-10 weeks; 25-30 g) received 1 × 10^11^ viral genome of AAV9-miR-200a or AAV9-miR-scramble by tail vein injection according to a previous study [17]. Four weeks later, male C57BL/6J mice received a single intraperitoneal injection of DOX-HCl (Adriamycin, Sigma-Aldrich, St. Louis, MO, USA) at 15 mg/kg to mimic acute DOX exposure according to a previous study [18]. Control mice were treated with the same volume of normal saline (NS), which was used to dissolve DOX. The animals were observed and weighed daily. Echocardiography was performed at 5 days after DOX injection, and animals were sacrificed thereafter. Knockdown of cardiac Nrf2 was carried out in the acute DOX regime experiment using adenoviral vectors carrying Nrf2 small hairpin RNAs (shRNAs) or scrambled shRNA, which was generated by Hanbio (Shanghai, China). To knock down Nrf2, mice were given an intramyocardial injection of 2 × 10^9^ viral genome particles in 1 location of the left ventricle according to a previous study [19]. One week after adenoviral injection, these mice were subjected to DOX injection to mimic acute DOX exposure. To mimic chronic DOX exposure, the mice in the DOX and DOX+miR-200a groups (*n* = 10 for each group) were injected intraperitoneally with DOX (5 mg/kg every week, the total cumulative dose is 20 mg/kg) for 4 times; the control mice received saline. After 2 weeks post the last injection, cardiac functions in the mice with chronic DOX injection were examined and the animals were then sacrificed. To observe the effect of miR-200a on the survival rate in mice with chronic DOX exposure, the mice (*n* = 15 for each group) were injected intraperitoneally with DOX (5 mg/kg every week, the total cumulative dose is 20 mg/kg) or saline for 4 times. After the first injection of DOX, these mice were observed daily for 6 weeks.

### 2.2. Echocardiography and Hemodynamic Analysis

Mice were exposed to mild anaesthesia with 1.5% isoflurane. Echocardiography was performed on six mice per group with the Vevo 2100 ultrasound, which was connected to an ultrasound system (SSD-5500; Aloka, Tokyo, Japan). Left ventricle end-diastolic and end-systolic diameters were measured.

To detect hemodynamic parameters, a microtip pressure-volume catheter (SPR-839; Millar Instruments, USA) was inserted through an apical stab into the ventricle to measure cardiac function. Hemodynamic measurements were analyzed using IOX software (EMKAtech).

### 2.3. Western Blotting

Proteins were extracted from frozen heart tissues and subjected to sodium dodecyl sulfate-polyacrylamide gel electrophoresis [20, 21]. After that, the proteins were transferred to PVDF membranes (Merck Millipore, Massachusetts, USA). Membranes were then blocked in nonfat milk for 2 hours and incubated overnight at 4°C with primary antibodies against Nrf2 (Abcam, Cambridge, MA, UK, ab62352, 1 : 1000), heme oxygenase 1 (HO-1, Abcam, ab13248, 1 : 1000), GAPDH (Abcam, ab181602, 1 : 1000), Bax (Abcam, ab32503, 1 : 1000), and Bad (Abcam, ab32445, 1 : 1000). After that, the membrane was reacted with the secondary antibodies for 1 h at room temperature, was stained with an enhanced chemiluminescence reagent, and was visualized using the BIO-RAD ChemiDoc Touch Imaging System (BIO-RAD, Hercules, CA, USA). GAPDH was used as the internal control.

### 2.4. RNA Extraction and Real-Time RT-PCR

Total RNA extraction was performed using a TRIzol reagent (Invitrogen Corp., CA). DNAse-treated total RNA (2 *μ*g) was reverse transcribed using SuperScript II reverse transcriptase (Invitrogen Corp.). Gene expression analysis was carried out using the Fast SYBR Green master mix (Applied Biosystems) and the QuantStudio 12k Flex real-time PCR system (ThermoFisher, Ecublens, Switzerland). Results were normalized to GAPDH mRNA as an internal control. The miRNA level was determined using a Bulge-Loop miRNA qRT-PCR Starter Kit (Ribobio Technology, Guangzhou, China). U6 was used as the internal control of miRNA.

### 2.5. Adult Cardiomyocyte Isolation and Mechanics Detection

Hearts were collected and mounted onto a temperature-controlled Langendorff system. Hearts were digested with a Ca^2+^-free Krebs-Henseleit bicarbonate buffer containing Liberase Blendzymes (0.1 mg/ml) for 30 min at 37°C, which was obtained from Roche Diagnostics (Indianapolis, IN). Adult cardiomyocytes with rod-shaped and clear edges were used in our detection within 6 h of isolation. An IonOptix™ soft-edge system (IonOptix, Milton, MA) was used to detect mechanical properties according to a previous study [22]. Cell shortening and relengthening, as reflected by peak shortening (PS) and maximal velocities of shortening/relengthening (±dL/dt), were assessed.

### 2.6. Glutathione, Malondialdehyde, and 4-Hydroxynonenal Detection

The fresh heart tissues were homogenized in ice-cold sodium phosphate buffer, and the supernatant fraction was collected for the detection of the levels of glutathione (GSH) and oxidized GSH (GSSG), malondialdehyde (MDA), and 4-hydroxynonenal (4-HNE). The kit for MDA detection was obtained from Nanjing Jiancheng Bioengineering Institute (Nanjing, China). A 4-HNE assay kit was provided by Abcam. The quantitative measurement of MDA and 4-HNE in heart homogenate was performed according to the manufacturer's instructions. GSH and GSSG were determined by a GSH and GSSG assay kit from Beyotime Biotechnology (Beijing, China) using the 2-vinyl pyridine spectrophotometric method.

### 2.7. Measurement of Cardiac Injury Markers

To detect plasma cardiac troponin I (cTnI) and the N-terminal probrain natriuretic peptide (NT-proBNP) levels, blood samples were collected from mice at 3 days after DOX injection. The NT-proBNP detection kit was provided by MyBioSource (CA, USA). The cTnI assay kit was obtained from Life Diagnostics, Inc. (West Chester, PA). NT-proBNP and cTnI were detected to reflect the acute cardiac injury according to standard procedures.

### 2.8. TUNEL Staining

Detection of apoptosis in the hearts was performed by a terminal deoxynucleotidyl transferase-mediated nick-end labelling (TUNEL) assay according to the instruction provided with the kit (Roche Diagnostics, Indianapolis). The nucleus was labelled with DAPI.

### 2.9. Cell Culture

H9c2 cells were cultured in DMEM (high glucose, Gibco) supplemented with 10% fetal bovine serum (FBS) and 1% penicillin and streptomycin. The H9c2 cells were pretreated with micrON miR-200a (50 nmol/l, Ribobio Technology) or micrON mimic negative control for 48 hours and then incubated with DOX at 5 *μ*g/ml or the same volume of PBS for 24 h. At the endpoint of experiments, cells were harvested for further detection.

The commercial rat Nrf2 siRNA and control siRNA were purchased from Santa Cruz Biotechnology (Santa Cruz, CA, USA). We used three siRNAs to deplete Nrf2 expression. The one that resulted in the most significant downregulation of endogenous Nrf2 expression as confirmed by PCR and western was used for further experiments. The H9c2 cells were seeded on 6-well plates at 1 × 10^5^ cells/well for 48 hours. After 60-70% confluence, the cells were transfected with control siRNA or siNrf2 (100 pmol/l) using Lipofectamine 2000 (Invitrogen Corp.). Cells were harvested after 48-hour transfection for further experiments.

### 2.10. DCF-DA Staining

Dihydrodichlorofluorescein diacetate (DCF-DA) staining was applied to measure the generation of ROS. H9c2 cells were reacted with DCF-DA (10 *μ*mol/l) for 30 min at 37°C in the dark. After washing 5 times with PBS, the cells were observed using a confocal microscope.

### 2.11. Statistical Analysis

Data were presented as means ± standard deviation. We used the unpaired *t*-test to compare significance between two groups. One-way ANOVA followed by the Tukey post hoc test was used to compare the difference between more than two groups. *P* values < 0.05 were considered to be statistically significant.

## 3. Result

### 3.1. miR-200a Was Decreased in Hearts of DOX-Treated Mice

**Figure 1:**
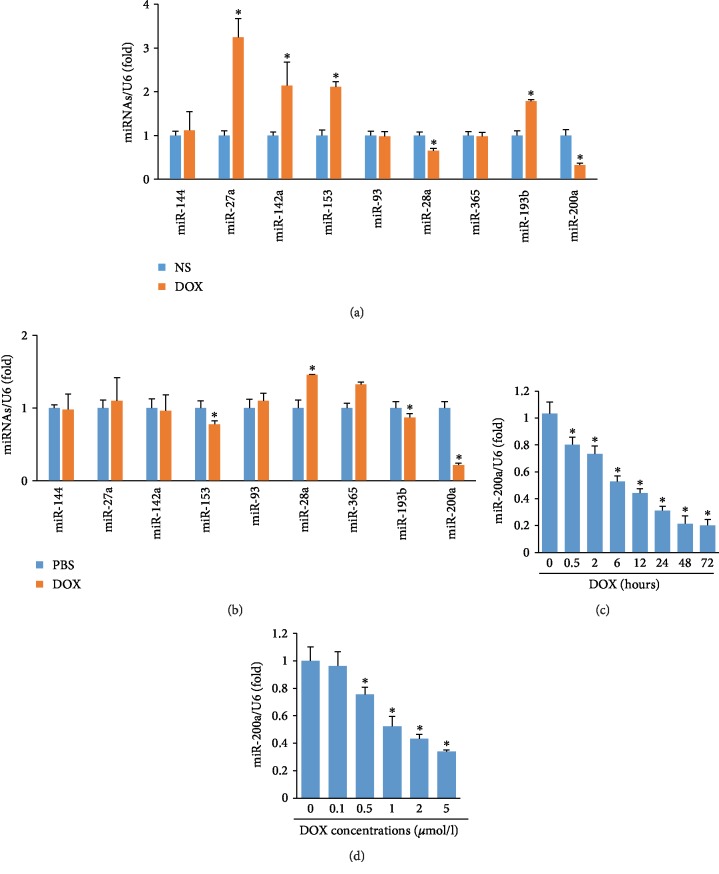
miR-200a was decreased in DOX-induced cardiac injury: (a) the levels of miRNAs in the hearts (*n* = 6); (b) the levels of miRNAs in the H9c2 cells (*n* = 6); (c, d) the levels of miR-200a in the H9c2 cells (*n* = 6). ^∗^*P* < 0.05 compared with the group with saline or PBS.

We first determined miRNAs that target Nrf2 in the mice with DOX injection. All the miRNAs that were reported to regulate the level of Nrf2 were detected [23], including miR-144, miR-27a, miR-142a, miR-153, miR-93, miR-28a, miR-365, miR-193b, and miR-200a. miR-27a, miR-142a, miR-153, and miR-193b expressions increased, while miR-28a and miR-200a expressions decreased in the hearts after DOX treatment (Figure 1(a)). Next, H9c2 cells were treated with DOX, and miRNAs that regulate the level of Nrf2 were also detected. miR-28a and miR-365 expressions increased, while miR-153 and miR-200a expressions decreased in the DOX-treated H9c2 cells (Figure 1(b)). Therefore, we concluded that miR-200a may be involved in DOX-induced cardiac injury. Further study revealed that miR-200a was significantly decreased in a time-dependent manner and dose-dependent manner (Figures 1(c) and 1(d)). These data suggested that miR-200a might play a key role in the DOX-induced cardiotoxicity.

### 3.2. miR-200a Overexpression Protected against DOX-Induced Acute Cardiac Injury

**Figure 2:**
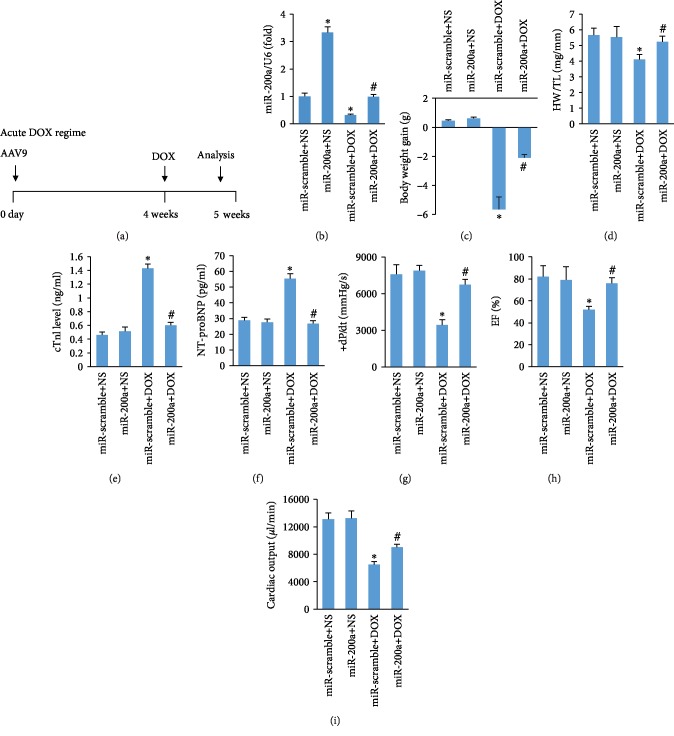
miR-200a overexpression improved cardiac function in mice: (a) schedule of the acute DOX regime experiment; (b) the levels of miR-200a in the hearts (*n* = 6); (c) the alteration in body weight (*n* = 12); (d) alterations in the ratio of heart weight to tibial length (*n* = 12); (e, f) the level of cTnI and NT-proBNP (*n* = 6), (g, h) the alteration in +dP/dt and EF in mice (*n* = 10); (i) cardiac output in the mice (*n* = 10). ^∗^*P* < 0.05 compared with the group with saline. ^#^*P* < 0.05 compared with mice after DOX injection.

We first established an acute model of DOX-induced cardiotoxicity. In this experiment, mice were first infected with AAV9-miR-200a or AAV9-miR-scramble for 4 weeks and were subjected to DOX or saline (Figure 2(a)). Reduced miR-200a expression in the hearts of DOX-treated mice was almost restored to the normal level (Figure 2(b)). The animals were then followed up for five days before sacrifice. miR-200a overexpression blunted the decrease of body weight in DOX-treated mice (Figure 2(c)). The ratio of heart weight to tibial length was significantly decreased in DOX-treated mice, and this pathological alteration was blocked (Figure 2(d)). A single injection of DOX (15 mg/kg) induced the elevation of cTnI and NT-proBNP level, which was prevented by miR-200a overexpression (Figures 2(e) and 2(f)). Administration of DOX caused worsening of left ventricle systolic function, as reflected by maximum first derivative of ventricular pressure with respect to time (+dP/dt), ejection fraction (EF), and cardiac output, but mice with miR-200a overexpression preserved +dP/dt, EF, and cardiac output compared with mice with miR-scramble after DOX treatment (Figures 2(g)–2(i)).

### 3.3. miR-200a Overexpression Reduced Adult Cardiomyocyte Contractile Dysfunction after DOX Treatment

**Figure 3:**
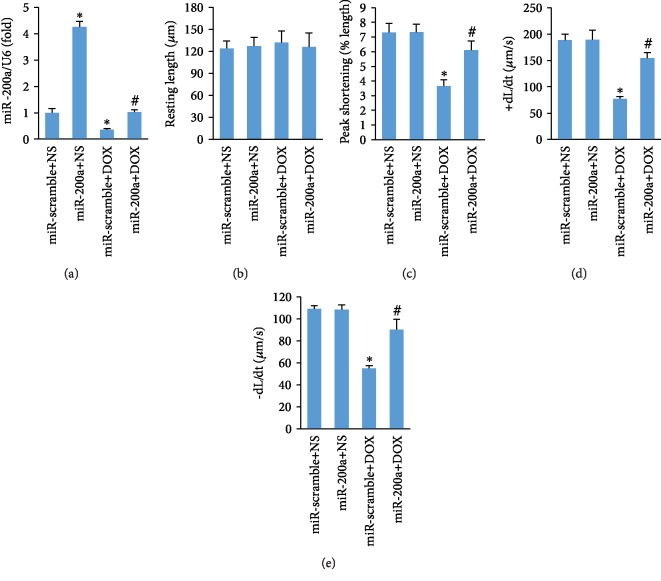
miR-200a improved adult cardiomyocyte contractile properties: (a) the levels of miR-200a; (b) resting cell length; (c) peak shortening; (d, e) maximal velocity of shortening (+dL/dt) and maximal velocity of relengthening (-dL/dt). ^∗^*P* < 0.05 compared with the group with saline. ^#^*P* < 0.05 compared with mice after DOX injection. *n* = 50 cells from 4 mice per group.

Subsequently, we determined the effect of miR-200a overexpression on contractile function of single adult cardiomyocyte which was isolated from mice with acute DOX injection. As expected, reduced miR-200a expression in the adult cardiomyocytes isolated from DOX-treated mice was restored to the normal level (Figure 3(a)). miR-200a has no significant effect on resting cell length between four groups (Figure 3(b)). Adult cardiomyocytes in the mice with DOX treatment showed a reduced peak shortening and maximal velocity of shortening/relengthening (±dL/dt). After miR-200a overexpression, these pathological alterations were largely blocked (Figures 3(c)–3(e)).

### 3.4. miR-200a Overexpression Attenuated DOX-Induced Oxidative Stress in Mice

**Figure 4:**
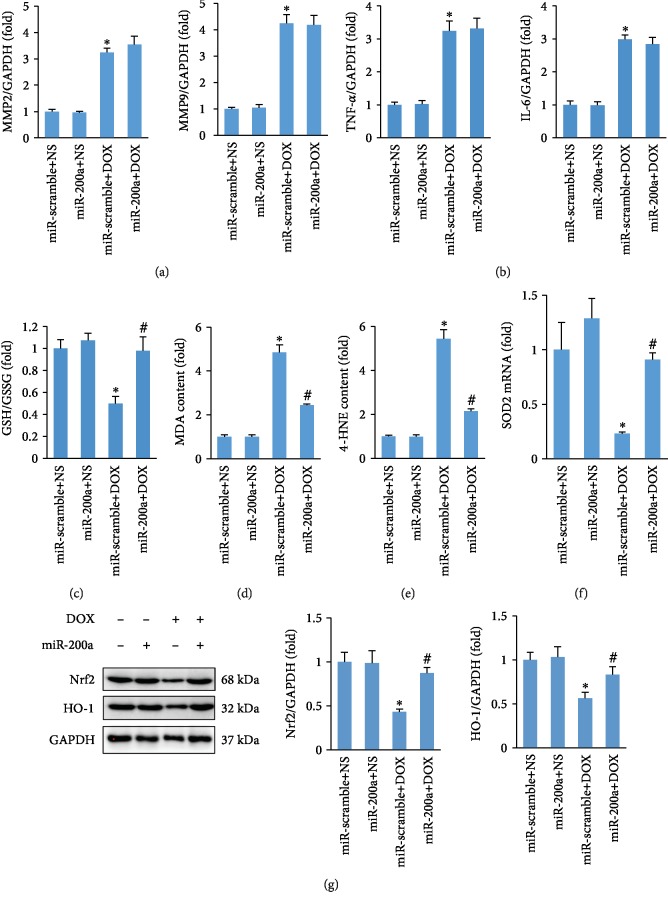
miR-200a reduced oxidative stress in DOX-treated mice: (a) the level of MMP2 and MMP9 in the hearts (*n* = 6); (b) the level of TNF-*α* and IL-6 in the hearts (*n* = 6); (c, d) the levels of GSH and cardiac MDA in the hearts (*n* = 6); (e) the level of 4-HNE in the hearts (*n* = 6); (f) the level of SOD2 mRNA in the hearts (*n* = 6); (g) protein expression of Nrf2 and HO-1 (*n* = 6). ^∗^*P* < 0.05 compared with the group with saline. ^#^*P* < 0.05 compared with mice after DOX injection.

Matrix metalloproteinase (MMP) activation is a key event in DOX-induced acute cardiotoxicity [24]. Therefore, we first detected the alteration in the mRNA level of MMP2 and MMP9 and found that miR-200a did not prevent the elevation of MMP2 and MMP9 expressions in DOX-treated mice (Figure 4(a)). Next, we detected the levels of inflammatory factors in DOX-treated hearts and found that there was no difference in the levels of inflammatory factors between miR-scramble+DOX and miR-200a+DOX groups (Figure 4(b)). Acute DOX injection decreased the ratio of GSH and GSSG, and this downregulation was blocked by miR-200a (Figure 4(c)). Acute DOX injection induced abnormal accumulation of 4-HNE and MDA, and these accumulations of 4-HNE and MDA were inhibited by miR-200a overexpression (Figures 4(d) and 4(e)). Further detection found that miR-200a restored the mRNA of SOD2 in DOX-treated mice (Figure 4(f)). Western blot analysis demonstrated that Nrf2 and its downstream target were markedly decreased in the DOX-treated group, and miR-200a almost restored Nrf2 and HO-1 protein expression to the normal levels (Figure 4(g)).

### 3.5. miR-200a Inhibited DOX-Induced Myocardial Apoptosis in Mice with Acute DOX Injection

**Figure 5:**
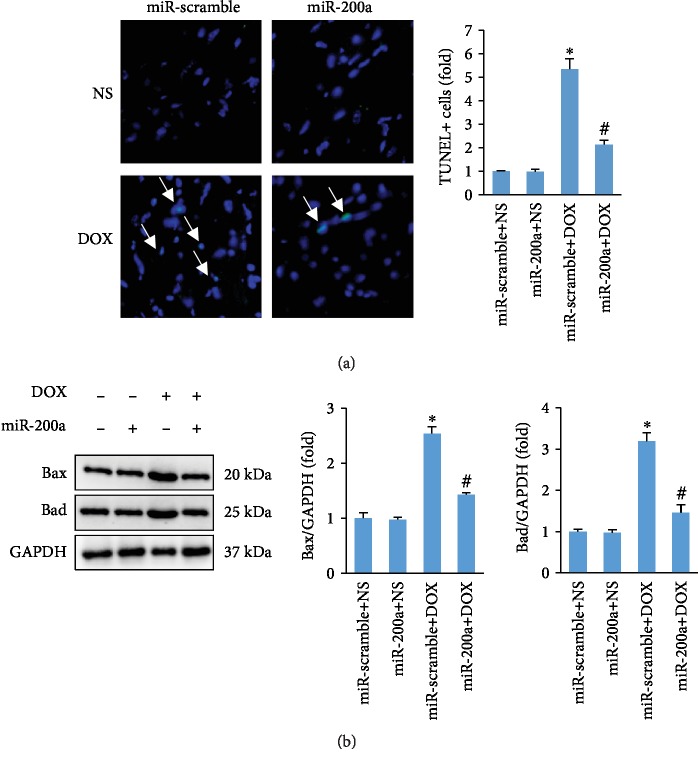
miR-200a suppressed cardiac apoptosis in DOX-treated mice. (a) TUNEL staining (*n* = 6). (b) Western analysis indicated the expression of Bax and Bad in the hearts (*n* = 6). ^∗^*P* < 0.05 compared with the group with saline. ^#^*P* < 0.05 compared with mice after DOX injection.

Expectedly, the number of TUNEL-positive cells was markedly increased in heart sections of mice with acute DOX compared to that in mice with saline only (Figure 5(a)). Interestingly, DOX-induced TUNEL-positive cells were attenuated by miR-200a (Figure 5(a)). Subsequent detection of Bax and Bad expressions in the hearts revealed that miR-200a attenuated the levels of Bax and Bad in mice (Figure 5(b)).

### 3.6. Nrf2 Deficiency Antagonized the Protective Effects against DOX-Related Injury in H9c2 Cells

**Figure 6:**
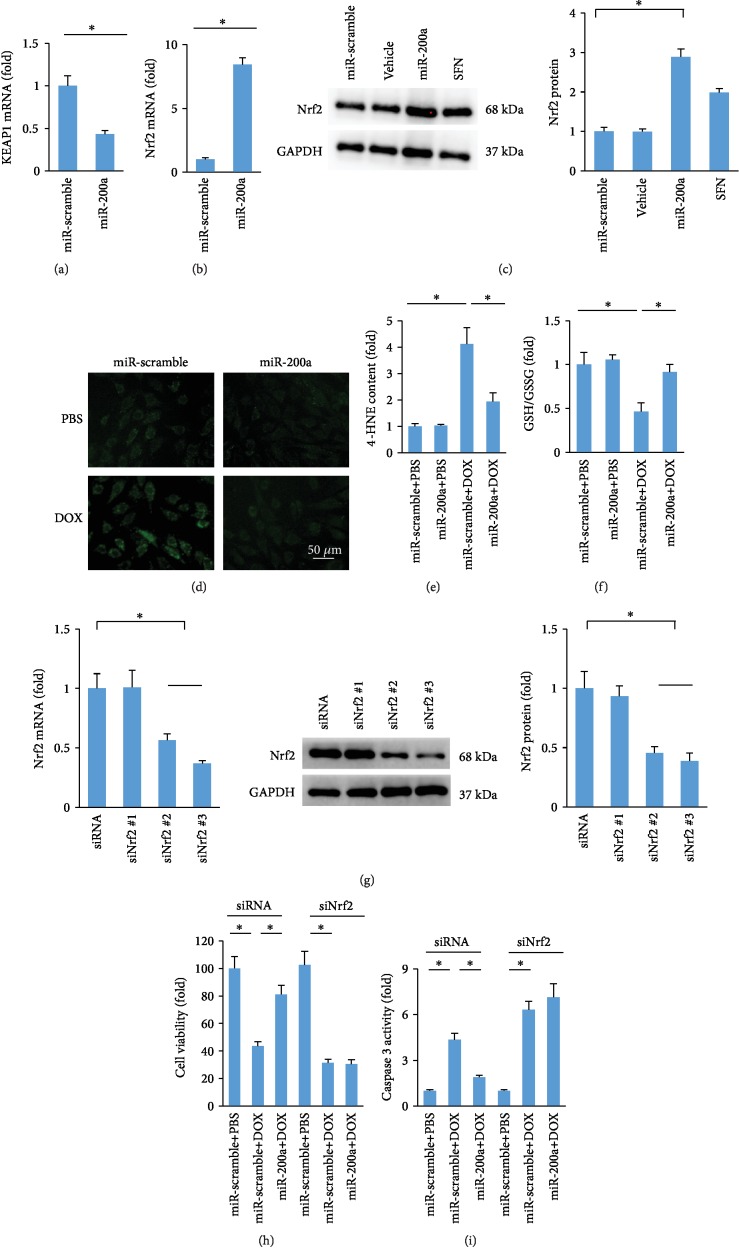
miR-200a provided cardioprotection via activating the Nrf2 signaling pathway: (a, b) the level of Keap1 and Nrf2 in the H9c2 cells (*n* = 6); (c) the protein expression of Nrf2 (*n* = 6); (d) DCF-DA staining; (e, f) the levels of 4-HNE and GSH in the cells (*n* = 6); (g) the level of Nrf2 in the cells (*n* = 6); (h) cell viability (*n* = 6); (i) caspase 3 activity (*n* = 6). ^∗^*P* < 0.05.

It has been reported that Keap1 interacted with Nrf2 and retained Nrf2 in the cytoplasm [8]. miR-200a destabilized Keap1 and resulted in a reduction in Keap1 protein level [16]. Thus, we measured Keap1 mRNA levels and found that Keap1 mRNA level was significantly decreased in miR-200a-infected cells (Figure 6(a)). Further analysis showed that miR-200a caused an increase in Nrf2 mRNA expression (Figure 6(b)). Consistent with this finding, we found that miR-200a overexpression also significantly increased Nrf2 protein expression (Figure 6(c)). We also confirmed that sulforaphane, a well-studied natural product, could increase Nrf2 protein expression (Figure 6(c)). Next, we compared the generation of ROS, as labelled by DCF-DA in DOX- and DOX+miR-200a-treated H9c2 cells. The data in our study showed that miR-200a could significantly block DOX-induced formation of ROS in H9c2 cells (Figure 6(d)). The increased 4-HNE content in cells with DOX treatment was also inhibited after miR-200a administration (Figure 6(e)). miR-200a also restored GSH/GSSG to the normal level in DOX-treated cells (Figure 6(f)). Next, we verified the hypothesis that the protection provided by miR-200a was mediated by Nrf2. We used three siRNAs to deplete Nrf2 expression. The one (siNrf2 #3) that resulted in the most significant downregulation of endogenous Nrf2 expression as confirmed by PCR and western was used for further experiments (Figure 6(g)). We found that miR-200a significantly improved cell viability and decreased caspase 3 activity in DOX-treated cells, and these protections were completely blocked after Nrf2 deficiency (Figure 6(h) and 6(i)).

### 3.7. The Protective Effects of miR-200a against DOX-Induced Acute Cardiotoxicity Were Reversed by the Deficiency of Nrf2 in Mice

**Figure 7:**
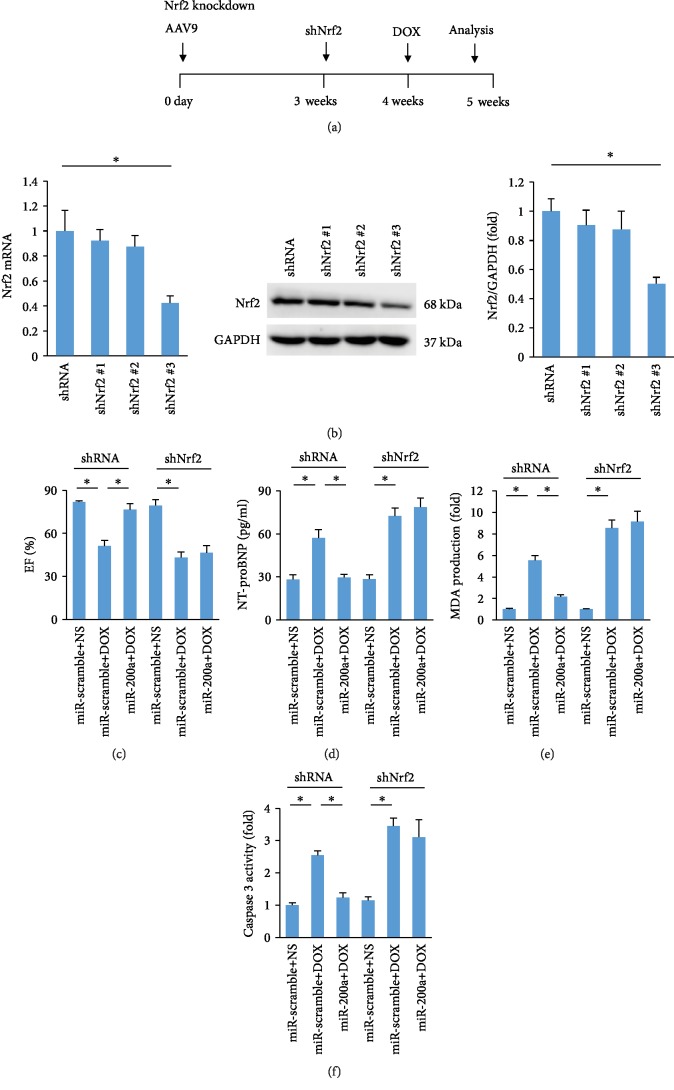
miR-200a could not provide cardiac protection against DOX-induced acute cardiotoxicity in Nrf2-deficient mice: (a) schedule of Nrf2 deficiency in mice with the acute DOX treatment; (b) the level of Nrf2 in the hearts (*n* = 6); (c) EF (*n* = 8); (d, e) the levels of NT-proBNP and MDA in the hearts (*n* = 6); (f) caspase 3 activity (*n* = 6). ^∗^*P* < 0.05.

To determine whether miR-200a exerted its protection via activation of Nrf2 in mice, we used three shRNAs to deplete Nrf2 expression in the hearts. The one (shNrf2 #3) that resulted in the most significant downregulation of endogenous Nrf2 expression as confirmed by PCR and western was intramyocardially injected (Figures 7(a) and 7(b)). One week after adenoviral injection, these mice were subjected to DOX injection to mimic acute DOX exposure. As indicated in our study, miR-200a lost its protection in cardiac injury, as reflected by EF, NT-proBNP, MDA content, and caspase 3 activity, in mice (Figures 7(c)–7(f)).

### 3.8. miR-200a Also Provided Cardiac Benefit in a Chronic Model of DOX-Induced Cardiotoxicity

**Figure 8:**
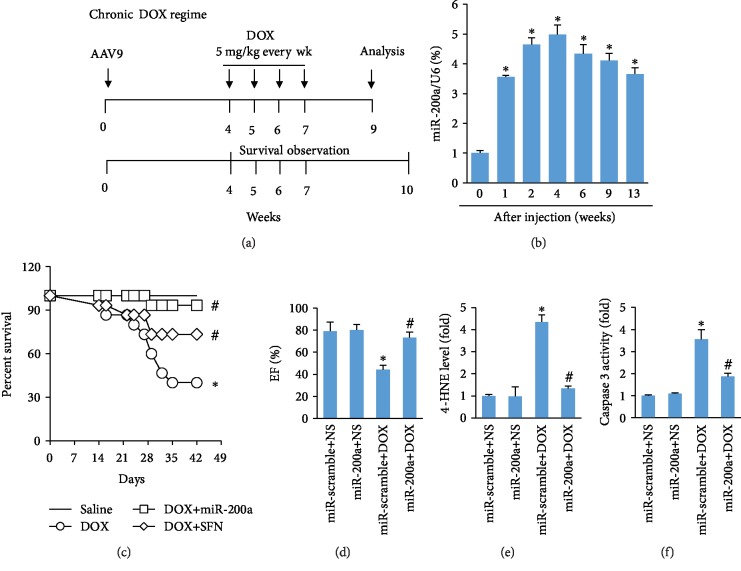
miR-200a protected the hearts from DOX-induced chronic cardiotoxicity: (a) schedule of the acute DOX regime experiment; (b) the level of miR-200a in the hearts (*n* = 6); (c) survival rate in the groups (*n* = 15); (d) EF in the four groups (*n* = 10); (e) the level of 4-HNE in the hearts (*n* = 6); (f) caspase 3 activity (*n* = 6). ^∗^*P* < 0.05 compared with the group with saline. ^#^*P* < 0.05 compared with mice after DOX injection.

To mimic chronic DOX exposure, the mice in the DOX and DOX+miR-200a groups were injected intraperitoneally with DOX (5 mg/kg every week, the total cumulative dose is 20 mg/kg) for 4 times (Figure 8(a)). As indicated, miR-200a was highly expressed even at 13 weeks after infection (Figure 8(b)). The survival rate in the DOX-treated group was significantly lower than that in the saline-treated group. Conversely, this was largely improved after miR-200a overexpression (Figure 8(c)). We also found that miR-200a was more effective than sulforaphane in chronic cardiotoxicity (Figure 8(c)). Chronic injection of DOX induced a significant decrease in EF, and this change was significantly attenuated in mice with miR-200a (Figure 8(d)). Chronic DOX administration was also associated with marked increases in myocardial 4-HNE and caspase 3 activity, and miR-200a overexpression largely attenuated these pathological changes in mice (Figures 8(e) and 8(f)).

## 4. Discussion

The death of cancer survivors was mainly attributed to cardiac factors [25], which emphasizes the need for pharmacological strategies offering protection against cardiotoxicity caused by anticancer drugs. In this study, we for the first time found that miR-200a supplementation could reduce cardiac injury, improve cardiac function, and attenuate DOX-related oxidative stress and cell apoptosis without affecting the level of MMP and inflammatory factors in mice. miR-200a also protected the hearts from DOX-induced chronic damage. These findings positively suggest that miR-200a overexpression strategies may be helpful for promoting cardiomyocyte survival in DOX-treated mice.

Recently, accumulating evidence supported the notion that miR-200a was closely involved in cardiovascular diseases [13, 23]. Yang et al. reported that miR-200a-5p promoted cardiomyocyte hypertrophy via inhibiting the expression of stress-related selenoproteins to alter glucose transport [26]. miR-200a-5p was identified to be upregulated under Se-deficient stimulation, and miR-200a-5p deficiency attenuated myocardial necroptosis induced by Se deficiency [15]. Inconsistent with the study, we found that miR-200a could provide protection against DOX-induced cardiac injury, as reflected by the body weight gain and the levels of NT-proBNP and cTnI. miR-200a supplementation also improved cardiac function in mice. We postulate that subtle differences in the animal strain and disease model might lead to discordant observations.

MMP2 and MMP9 mRNAs were significantly increased in the ventricles of mice at 2 days after DOX treatment [24]. Consistent with this finding, we also confirmed that MMP2 and MMP9 were increased in the mouse hearts after treatment with DOX. However, there was no difference between DOX+miR-scramble and DOX+miR-200a groups in the mRNA levels of MMP2 and MMP9, implying that miR-200a exerting its function was not mediated by the alteration in MMP content. Inflammation also played a key role in the pathogenesis of DOX-related cardiac injury [27]; therefore, we detected alteration in the mRNA levels of inflammatory factors. The data in our study suggested that miR-200a cannot affect the cardiac inflammation in the DOX-treated mice.

It has been reported that scavenging ROS protects against DOX-induced cardiac apoptosis [28]. Cardiac-specific overexpression of metallothionein protected against DOX-related cardiac dysfunction [29]. Here, we found that 4-HNE and MDA productions were enhanced in the DOX heart and were reduced by miR-200a overexpression. In addition, we have also found that miR-200a preserved the reduced GSH level induced by DOX but also elevated to a level comparable to that of normal control mice. Further detection found that miR-200a also largely attenuated DOX-induced cell apoptosis in hearts and in H9c2 cells. As expected, we found that miR-200a could activate Nrf2 and miR-200a lost its protection against oxidative stress and cell viability after Nrf2 deficiency, suggesting that the protection of miR-200a was dependent on the activation of Nrf2.

Several natural products have been evaluated for their ability to attenuate DOX-induced cardiotoxicity but with little success [30, 31]. Low bioavailability and low scavenging efficacy toward oxidants were the main reasons [32]. In our study, we compare the ability of sulforaphane and miR-200a to activate Nrf2 in vitro and in vivo and found that the two both largely activate Nrf2. Moreover, miR-200a had a better effect on the survival rate in chronic cardiotoxicity. In addition, several lines of evidence demonstrated that miR-200a was a tumor suppressor [33, 34], suggesting that miR-200a treatment might not compromise the oncological efficacy of DOX.

In conclusion, our results suggest that miR-200a protects DOX-induced acute toxicity by activating Nrf2 to attenuate oxidative stress and apoptotic cell death, prevents DOX-induced cardiomyopathy, and ameliorates cardiac dysfunction. The present findings suggest that miR-200a supplementation may represent a new cardioprotective strategy against DOX-induced cardiotoxicity.

## Data Availability

The data that support the findings of this study are available from the corresponding author upon reasonable request.
